# 1809. Treatment Outcomes in Piperacillin-tazobactam Non-susceptible (TZP-NS)/Ceftriaxone Susceptible (CRO-S) Infections

**DOI:** 10.1093/ofid/ofac492.1439

**Published:** 2022-12-15

**Authors:** Yanina Dubrovskaya, John Cao, Justin Siegfried, Arnold Decano, Dana Mazo, Sarah Hochman, Ioannis Zacharioudakis, Sadie Solomon, John Papadopoulos

**Affiliations:** NYU Langone Health, New York, New York; NYU Langone Health, New York, New York; NYU Langone Health, New York, New York; NYU Langone Health, New York, New York; New York University, New York University, New York; NYU Langone Health, New York, New York; New York University, New York University, New York; NYU Langone Health, New York, New York; NYU Langone Health, New York, New York

## Abstract

**Background:**

Piperacillin-tazobactam-non-susceptible/ceftriaxone-susceptible (TZP-NS/CRO-S) *Escherichia coli* (*Ec)* and *Klebsiella pneumoniae* (*Kp)* phenotypes are hypothesized to be due to hyperproduction of TEM-1/2 and SHV-1 penicillinases. Limited available literature evaluating treatment outcomes in patients with TZP-NS/CRO-S infections may lead to unnecessary carbapenem use.

**Methods:**

This was a retrospective study of non-critically ill adults hospitalized between 2013-2021, and treated for at least 48 hours for TZP-NS/CRO-S *Ec* or *Kp* infections. Patients with concomitant multi-drug resistant gram-negative infections were excluded. The primary composite endpoint included the need for escalation to intensive care unit (ICU), infection- or treatment-related readmission, mortality, and infection recurrence. Outcomes were compared between groups who received carbapenem (CG) vs carbapenem-sparing agents (CSG) as targeted therapy.

**Results:**

Out of 1062 patients screened, 200 were included, with 51 in the CG and 149 in the CSG. Baseline characteristics, including Charlson Comorbidity Index (6 [IQR 3-10] vs 6 [4-9], p=0.64), were similar between groups, except for more immunocompromised patients in the CG (29% vs 11%, p=0.001). The most common infection sources were urinary (31% vs 57%, p=0.002), bloodstream (BSI) (18% vs 17%, p=0.887) and intra-abdominal (IAI) (20% vs 8%, p=0.023). Eighty-eight percent of the CG received meropenem, while the most common targeted therapy in the CSG was ceftriaxone (58%) followed by cefepime (24%). The primary composite endpoint was numerically higher in the CG (27% vs 17%, p=0.123), with no differences based on infection type (urine 25% vs 18%, p=0.495; BSI 33% vs 16%, p=0.348; IAI 40% vs 33%, p=1). More patients in the CSG were switched to oral therapy (15 [29%] vs 100 [67%], p< 0.001), with numerically 2 days shorter time to oral switch (6 [3-9] vs 4 [3-7] days, p=0.196). Out of those switched to oral therapy, most had a urinary source (8/15 [53%] vs 59/100 [59%]).

**Conclusion:**

Our study did not find better clinical outcomes with carbapenem therapy for TZP-NS/CRO-S infections, thus CS agents may be considered to spare carbapenems in non-critically ill patients. Further studies are warranted to compare these outcomes in the critically ill.

**Disclosures:**

**All Authors**: No reported disclosures.

